# LRBA in the endomembrane system

**DOI:** 10.25100/cm.v49i2.3802

**Published:** 2018-09-30

**Authors:** Catalina Martínez Jaramillo, Claudia M. Trujillo-Vargas

**Affiliations:** Grupo de Inmunodeficiencias primarias, Facultad de Medicina, Universidad de Antioquia UdeA, Medellín, Colombia.

**Keywords:** Primary immunodeficiencies, LRBA deficiency, vesicle trafficking, autophagy, apoptosis, Inmunodeficiencias primarias, deficiencia de LRBA, tráfico vesicular, autofagia, apoptosis

## Abstract

Bi-allelic mutations in *LRBA* (from *Lipopolysaccharide-responsive and beige-like anchor protein*) result in a primary immunodeficiency with clinical features ranging from hypogammaglobulinemia and lymphoproliferative syndrome to inflammatory bowel disease and heterogeneous autoimmune manifestations. LRBA deficiency has been shown to affect vesicular trafficking, autophagy and apoptosis, which may lead to alterations of several molecules and processes that play key roles for immunity.

In this review, we will discuss the relationship of LRBA with the endovesicular system in the context of receptor trafficking, autophagy and apoptosis. Since these mechanisms of homeostasis are inherent to all living cells and not only limited to the immune system and also, because they are involved in physiological as well as pathological processes such as embryogenesis or tumoral transformation, we envisage advancing in the identification of potential pharmacological agents to manipulate these processes.

## Resumen

Las mutaciones bi-alélicas en *LRBA* (del inglés, *Lipopolysaccharide-responsive and beige-like anchor protein)* conllevan a una inmunodeficiencia primaria con características clínicas que abarcan desde hipogamaglubulinemia y síndrome linfoproliferativo hasta una enfermedad inflamatoria intestinal y manifestaciones autoinmunes heterogéneas. Se ha demostrado que la deficiencia de LRBA afecta el tráfico vesicular, la autofagia y la apoptosis pudiendo generar alteraciones en la regulación de varios procesos importantes para la inmunidad. En esta revisión discutiremos la relación de LRBA con el sistema endovesicular en el contexto del tráfico de receptores, la autofagia y la apoptosis. Estos mecanismos de homeostasis son inherentes a todas las células y no están limitados a las células del sistema inmune, están involucrados en procesos fisiológicos y patológicos, como la embriogénesis o la transformación tumoral. El entendimiento de la función de LRBA permitirá avanzar en la identificación de los posibles blancos farmacológicos para manipular estos procesos.

## Introduction

More than 400 genes have been reported to affect the immune system either quantitative and/or qualitatively and also, in a symptomatic manner ^1^ ^.^ Among them is included *LRBA* (*Lipopolysaccharide-responsive and beige-like anchor protein*); either homozygous or compound heterozygous mutations in this gene cause loss of LRBA protein expression, which is associated with a broad spectrum of clinical and immunological manifestations ^1^. The first report on LRBA deficiency was performed in five patients from four consanguineous families with hypogammaglobulinemia, autoimmune manifestations and recurrent infections ^2^.^.^ Subsequently, several reports of individuals with LRBA deficiency have been published, who suffer from a wider spectrum of clinical manifestations including: increased susceptibility to infections, polyautoimmunity, lymphoproliferation and gastropathy, all associated or not with hypogamaglobulinemia ^3^ ^-^ ^5^. Moreover, an asymptomatic individual has also been reported ^6^.

With regard to the immunological findings, T lymphocytes from patients with the LRBA deficiency presented with an increase in either starvation- or staurosporine-induced and controversially, a decrease in Fas-induced apoptosis (Staurosporine is a potent inhibitor of protein kinases and activator of caspases) ^7^. Furthermore, a decrease in the expression of CTLA-4 (Cytotoxic T-Lymphocyte Antigen 4) was described in these cells, both in the surface membrane and intracellularly ^8^. In B cells from patients, a decrease in the *in vitro* production of antibodies was observed, together with an increase in apoptosis and a defect in the starvation-induced autophagy ^2^. Taken together, these studies suggest that LRBA is important for the humoral response, immune regulation and defense against infections, both for extracellular and intracellular microorganisms, influencing the survival and homeostasis of T and B cells.

## LRBA: general characteristics

**Figure 1:**
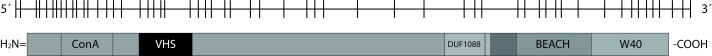
Schematic representation of LRBA and the coding protein. Human LRBA contains 750,839 bp spanning 58 exons. Its cytogenetic location is 4q31.3. The human LRBA protein comprises 2,863 amino acids and is composed of multiple domains. The predicted molecular mass is 319 kD.

*LRBA* is located on chromosome 4q31.3 and comprises 750,839 bp along 58 exons. The protein contains 2,863 amino acids and has a molecular mass of (319 kDa (Fig. 1). Three different transcripts of 1344, 836 and 787 bp has been detected in different mice tissues ^9^. Additionally, the expression of the LRBA messenger RNA (mRNA) increases 2-4 times after stimulation with lipopolysaccharide (LPS) in different cell lines ^9^.

This protein has different domains and among them, the BEACH domain (beige and Chediak-Higashi) is the most characterized. This domain, with (280 amino acids in length, is conserved in several proteins grouped overall as the BEACH domain containing proteins (BDCP) ^10^, and is mostly located towards the C-terminal ^11^ ^,^ ^12^. The function of this domain is unknown and the sequence does not show homology with any other protein in the human proteome database ^11^ ^,^ ^12^. In addition to the BEACH domain, the BDCP shares the ConA-like domain (concanavalin A-like), PH (Pleckstrin homology) and WD40 (repeats of tryptophan and aspartic acid) domains. On the other hand, the domain DUF1088 (domain of unknown function 1088) is shared only by LRBA and its paralog neurobeachin, which may indicate specific functions of these two proteins not shared by other members of the BDCP.

In addition to these domains, LRBA seems to contain a VHS domain (VPS (vacuolar protein sorting)-27, Hrs (hepatocyte growth factor-regulated tyrosine kinase sustrate) STAM (signal transducing adaptor molecule) domain) ^13^. 

The possible functions of all these domains are explained in Table 1.

**Table 1:** General characteristics of the LRBA protein domains.

Domain	Characteristics	Reference
BEACH	It is present in proteins involved in vesicle trafficking, membrane dynamics and receptor signaling	^12^ ^,^ ^13^
PH	Interacts with phosphatidyl-inositol within biological membranes, hetero-trimeric G proteins and protein kinase C. This allows the appropriate targeting of proteins to different cellular compartments or the activation of signal transduction pathways.	^54^
WD40	Involved in signal transduction, cell cycle control and apoptosis. It allows transient protein-protein interaction or the assembly of protein complexes.	^55^
ConA-like	Participates in trafficking and classification of proteins along the secretory pathway	^56^
DUF1088	The function of this domain is unknown. However, a penta-arginine sequence located in the DUF1088 domain of NBEA was identified as a potential signal of nuclear localization	^57^
VHS	Located in the N-terminal portion of proteins associated with endocytosis and/or vesicular trafficking. It likely functions as an adapter domain that helps to locate proteins in the cell membrane or in membranes of the endocytic machinery	^13^

With respect to tissue-specific expression, transcripts of this protein have been observed mainly bone marrow, lymph nodes, spleen, fetal liver, placenta, kidney and pancreas by RT-PCR (Reverse transcription polymerase chain reaction) and qPCR (quantitative polymerase chain reaction) ^2^. Remarkably, the LRBA expression is not limited to the immune system, and considerably increases in several cancer tissues ^14^. These findings suggest that LRBA function is more related to basic cellular mechanisms of growth, development and survival. At the subcellular level, using a probe directed against the LRBA BEACH domain, this protein has been observed in the cytosol, Golgi apparatus and some lysosomes in the LPS-stimulated murine macrophage cell line RAW264.7 ^9^. In addition, with the use of the electron microscopy, LRBA is observed in endoplasmic reticulum, plasma membrane and clathrin-associated endocytic vesicles in the same cell line ^9^.

## LRBA function

### LRBA participates in vesicular trafficking and receptor recycling

CTLA-4 (Cytotoxic T-Lymphocyte Antigen 4) is a protein receptor located in the plasmatic membrane of activated T cells. It functions to regulate homeostasis and peripheral immunological tolerance through the inhibition of T cell activation, competing with the costimulatory molecule CD28 for the specific ligation of CD80 and CD86, which are expressed on the surface of antigen-presenting cells ^15^. While CD28 resides mainly on the cell surface, CTLA-4 is located first in intracellular compartments such as Golgi apparatus, endosomes, secretory granules and lysosomes. The T cell receptor (TCR) stimulation promotes CTLA-4 trafficking from vesicular compartments to the cell membrane, however, once it is already in the membrane, this receptor is continuously endocytosed via clathrin-coated vesicles. After endocytosis, some CTLA-4 molecules are recycled to the cell membrane while others are rapidly degraded in lysosomal compartments ^16^ ^,^ ^17^. CTLA-4 contains a small cytoplasmic tail with two tyrosine motifs at position Y201VKM and Y218FIP. Several intracellular proteins bind to the Y201VKM motif, including AP-1 and AP-2 (clathrin-associated adapter). AP-2 mediates the internalization of CTLA-4 from the cell surface to endosomes and lysosomal compartments whereas AP-1 regulates CTLA-4 trafficking from the Golgi apparatus to endosomal compartments and lysosomes for degradation ^17^. In a recent study, it was demonstrated that LRBA is necessary for the vesicular trafficking of CTLA-4 towards the plasma membrane ^8^. LRBA regulates the recycling of CTLA-4 from endosomes to the cell membrane supporting the maintenance of its intracellular levels for the immediate mobilization to the surface membrane, as this molecule is required. Thus, a deficiency of LRBA favors a rapid degradation of CTLA-4 in the lysosome compartments, with its concomitant decrease both intracellular and extracellularly ^8^. This work also demonstrated that LRBA interacts through its ConA-like- and the PH-BEACH domains with the cytoplasmic tail of CTLA-4, specifically with the Y201VKM motif. Finally, the silencing of AP-1, but not AP-2 partially rescues the CTLA-4 loss in the LRBA deficient cells. These results suggest that LRBA blocks the trafficking of CTLA-4 to lysosomes, competing with AP-1 for binding to its specific binding motif ^8^. Recent findings indicated that Foxp3 + CD4 + cells from LRBA-deficient mouse spleen cells also exhibit an intracellular decrease in CTLA-4 ^18^.

Other study that demonstrates the LRBA role in vesicular trafficking is related to the epidermal growth factor receptor (EGFR). Dominant-negative *LRBA* mutants decreases the expression and phosphorylation of EGFR ^14^. EGFR is a tyrosine kinase receptor that is expressed in a wide variety of tissues, interacting not only with EGF but also with TGF-β (Transforming growth factor-β), and other ligands. This induces the dimerization and trans-auto-phosphorylation of the receptor by recruiting proteins that activate signaling pathways necessary for the proliferation, differentiation, cell growth, migration and inhibition of apoptosis ^19^. Similar to CTLA-4 in lymphocytes, the EGFR expression on the cell surface is regulated by clathrin-mediated endocytosis, which leads either to its degradation via the lysosome compartment or its recycling to the cell surface ^20^ ^,^ ^21^. Defects in the vesicular trafficking of this receptor result in an aberrant localization, which enhances signaling, allowing the development of cancer ^22^. Although it is still not known if LRBA and EGFR interact physically, it has been demonstrated that AP-2 facilitates endocytosis and trafficking of EGFR by the endocytic route ^3^ ^.^

Finally, LRBA deficient patients exhibited a decrease in apoptosis mediated by the death receptor Fas, as compared to healthy donors ^6^. Fas receptor promotes apoptosis through the extrinsic pathway, after binding to Fas ligand (FasL). After this binding, a process of FasL internalization occur, similar to EGFR, which is crucial to trigger apoptosis ^24^. To date, it is unknown whether LRBA-deficient patients exhibit alterations in the amount of FasL in the plasmatic membrane, but the increase in serum FasL in these patients could suggest that the LRBA deficiency may have an effect on FasL, similar to CTLA-4, regulating its trafficking either towards plasmatic membrane or lysosomes ^25^. 

These evidences suggest that LRBA might be involved in the vesicular trafficking of other receptors in lymphocytes. In LRBA deficient mice, defects have been reported in the phosphorylation of ERK1/2 (extracellular signal-regulated kinases) and AKT in natural killer (NK) cells stimulated with anti-NKG2D or anti-NKp46 antibodies, with a significant decrease in the production of IFN-gamma by these cells after stimulation ^26^. Although this study did not determine the expression levels of these receptors in LRBA-deficient mice, there is some evidence that the exposure to specific ligands induces the degradation of NKG2D and DAP10, the intracellular adapter molecule of this receptor in lysosomes ^27^. This regulation might be mediated by clathrin ^28^. It is not known if LRBA is involved in the intracellular trafficking of these receptors in NK cells. Likewise, considering that the LRBA deficiency leads to defects in antibody responses, it would be also important to consider whether this molecule modulates the expression and function of other receptors whose expression is modulated by vesicular compartments, especially in regard to B cells activation ^29^.

Despite these studies on the LRBA-mediated modulation of some cellular receptors, little is known about the intracellular pathways that this molecule employ to perform this function. This transport seems to be carried out by the recruitment of proteins in clathrin-coated vesicles, on which the receptors are removed to early endosomes and subsequently degraded by lysosomal enzymes or recycled to the cell surface ^30^. AP-1 is considered necessary for this trafficking. The location of AP-1 in the Golgi apparatus and endosomes depends on its direct binding to Arf (ADP-ribosylation factor) -1 and specifically in recycling endosomes, to Arf-6, among other protein complexes ^31^. As already mentioned, LRBA inhibits the degradation of CTLA-4 in lysosomes by competition with AP-1, which suggest that this protein is necessary for mobilization to these compartments ^8^. However, the relationship of LRBA with the endosomal pathway is unclear. The recycling of receptors also depends on vesicular compartments, and the proteins this required, namely, Arf, enzymes such as phospholipase D and Rab proteins. Interestingly, a study conducted in *Drosophila* suggests a possible functional interaction between bchs (blue cheese), a member of the BEACH family, and Rab11 ^32^. These findings suggest that, as well as Rab11, Bchs could be involved in the regulation of vesicle trafficking.

Therefore, studies quantifying the expression of the mentioned receptors in LRBA deficient cells (either by silencing or from patients) are needed. It would also be useful to use advanced microscopy techniques to investigate the location of LRBA in other organelles involved in this type of trafficking. In addition, studies of co-immunoprecipitation between LRBA and vesicular trafficking proteins, the use of drugs that block this trafficking or the implementation of negative dominant ARFs, phospholipase D or Rab, could expand our knowledge about the interaction of LRBA with other vesicular trafficking molecules and thus determine its relationship with the membrane expression of receptors such as EGFR, Fas or the activating receptors of NK cells, B cells or others in immune cells. 

### LRBA is a molecule involved in autophagy

Autophagy is a mechanism of lysosomal degradation by which the cell destroys damaged organelles and microorganisms and helps to recycle macromolecules. In this process, a double membrane of isolation or phagophore is formed near to the target, which is then elongated, surrounding it, to later mature with the assimilation of a cytosolic charge forming the autophagosome. For the maturation to be effective, the autophagosome must be fused with early or late endosomes forming a structure called amphisome ^33^ ^,^ ^34^. Finally, the mature autophagosomes fuses with lysosomes to proceed with the degradation of its content by lysosomal proteases and hydrolytic enzymes ^35^. 

Several of the genes homologous to LRBA are involved in autophagy. WDFY3 (WD and FYVE zinc finger domain containing protein 3) encodes a protein that colocalizes with autophagosome markers ^36^ ^,^ ^37^. In addition, the PH-BEACH domain interacts with the p62 protein ^34^. P62 is a protein located in the autophagosome formation sites and is associated with both LC3 (microtubule-associated protein 1A / 1B-light chain 3) and ubiquitinated proteins ^34^ ^,^ ^38^ ^,^ ^39^. LC3 is a cytosolic protein that when conjugated with a phosphatidylethanolamine is recruited to the autophagosome membrane favoring the autophagic influx ^34^. On the other hand, defects in the gene *mauve* (mv) in *Drosophila*, which is ortholog of *LYST*, affect the autophagosome function ^40^.

In immortalized B cells from patients with LRBA deficiency, a significant reduction in the autophagy influx in response to starvation, together with an increase in the Golgi apparatus area, accumulation of autophagosomes and the presence of centrioles is observed, in comparison with those cells from healthy individual ^2^. These results suggest that the autophagy influx is impaired in the LRBA deficiency, a finding that was demonstrated by a deficient fusion of autophagosome to lysosomes in immortalized B cells from LRBA-deficient patients subjected to starvation ^2^. However, what are the possible functions of LRBA in the autophagy influx? Considering that both, the cell surface receptor trafficking and autophagy need endocytic vesicles, and that LRBA is likely located in these vesicles, a possible hypothesis would be that LRBA facilitates fusion between autophagosomes and the late endosomes, and therefore, the formation of the amphisome. One of the markers of late endosomes is Rab7 and this molecule is required for the formation of amphisomas in CHO cells transfected with the dominant negative mutant of Rab7, T22N ^33^. Another hypothesis is that, in the same manner as the CTLA-4 vesicular trafficking, also in the context of autophagy in lymphocytes, LRBA would compete with clathrin adapter proteins, regulating this process. This since molecules such as clathrin and its adapter proteins are also important for the autophagic influx ^40^ ^-^ ^42^. On the other hand, *in silico* tools have predicted that LRBA interacts with LC3 ^13^. 

In an attempt to associate the defects in autophagy with the clinical characteristics found in LRBA deficiency individuals, it is important to bear in mind that one of the most common clinical manifestations among these individuals is the gastropathy, phenotypically expressed in many cases as Inflammatory Disease Intestinal (IBD) or IBD-like disease, a condition characterized by recurrent destructive inflammation in the intestinal tract ^3^ ^-^ ^5^ ^).^ An increase in the production of IL-1β in the interstitial mucosa has been implicated with uncontrolled inflammation in the course of this disease ^43^. Macrophages derived from *ATG16L1* (autophagy-related 16-like 1)-knockout mice present with a defect in autophagy and exhibit a high production of IL-1β after LPS stimulation ^44^. Likewise, the polymorphism rs2241880 in *ATG16L1* increased the production of IL-1β in peripheral blood mononuclear cells isolated from patients with IBD ^45^. Autophagy can regulate the production of IL-1β through two pathways: in the absence of autophagy, defective mitochondria accumulate generating an increase in ROS (reactivating oxygen species) and releasing mitochondrial DNA into the cytoplasm, events that further activate the inflammosome, increasing the production of IL-1β ^46^ ^,^ ^47^. On the other hand, the degradation of pro-IL-1β mediated by autophagy processes limits the substrate available for the inflammasome, thus regulating the activation of IL-1β ^48^ ^,^ ^49^. Therefore, alterations in autophagy would impair this mechanism, increasing the levels of IL-1β and inducing inflammation. It is not known if the intestinal manifestations in LRBA deficient patients are mediated by an excessive activity of the inflammosome and exaggerated production of IL-1β.

Microscopy studies with LRBA deficient cells are required to study the intracellular distribution of molecules such as Rab7 and LC3 after the autophagic influx. It would also be interesting to investigate if LRBA interacts physically with any of the Rab molecules involved in the different types of autophagy, by means of co-immunoprecipitation assays. The role of LRBA in inflamosome activity could be addressed by measuring the production of IL-1β in LRBA deficient phagocytic or intestinal epithelial cells or by determining the activity of active auto-inflammosome by means of redistribution of ASC (apoptosis-associated speck-like protein containing a caspase-recruitment domain), which is a protein that recruits the necessary caspases for the activation of the inflammosome. 

### LRBA participates in apoptosis.

In LRBA deficient patients’ immortalized B cells subjected to starvation, a significant increase in apoptosis is observed ^2^. Additionally, in *LRBA-*knockdown HeLa cells exposed to ultraviolet light or staurosporine, a greater cleavage of PARP (Poly (ADP-ribose) polymerase) and caspase 3 are observed, events that are necessary to initiate the cascade of signaling that eventually leads to apoptosis ^14^. 

Apoptosis is a type of programmed cell death to control development and growth. For long time, caspases have been considered as the proteases involved in the execution of the apoptosis program, and nowadays it is known that the partial destabilization of the lysosomal membrane, with the consequent release of hydrolytic enzymes, can initiate and execute apoptosis, byactivating caspases ^50^ ^,^ ^51^. In germinal centers derived from tonsils, it is observed that B cell lysosomal destabilization contributes to apoptosis ^50^. The induction of lysosomal damage in these cells resulted in the cleavage of caspase 8 and also of DNA, due to the increased activity of cathepsin B in the cytoplasm. Interestingly, it has been observed that p53 induces lysosomal destabilization ^52^ and the LRBA promoter is negatively regulated by p53 ^14^. p53 is a tumor suppressor gene that encodes a nuclear transcription factor, whose expression increases in response to damage, blocking the cell cycle either to facilitate DNA repair or to induce apoptosis ^52^. It is possible to hypothesize that, p53 would induce lysosomal destabilization in the absence of LRBA, and therefore, trigger apoptosis in lymphocytes. However, the mechanism by which p53 induces lysosomal destabilization or how LRBA protects cells from apoptosis is up to date unknown.

However, a complex crosstalk between autophagy and apoptosis exists, that is critical for cell fate. Under certain cellular conditions, autophagy can promote cell survival and prevent apoptosis ^53^. For example, increased autophagy in cells deprived of nutrients or growth factors allows cell survival by inhibiting apoptosis. A suggested mechanism by which autophagy inhibits apoptosis is the sequestration of mitochondria damaged by the autophagosome, thus preventing the released mitochondrial cytochrome c to form a functional apoptosome in the cytoplasm ^53^ ^.^ However, in other conditions, autophagy may culminate in cell death, dependently or independently of apoptosis ^53^. Additionally, there are several proteins that share a dual role in both apoptosis and autophagy ^53^. These autophagic proteins positively or negatively regulate mitochondrial apoptosis directly or indirectly through the cleavage of proteases ^54^. In the context of LRBA deficient cells, defective autophagy together with an increase in apoptosis has been demonstrated. It is not known if either alterations in the clearance of damaged mitochondria or an exaggerated cleavage of proteases are responsible for this cellular phenotype.

Therefore, it is necessary to evaluate the quantity and integrity of the lysosomes in LRBA deficient immune cells. In addition, the kinetics of lysosomal destabilization could be estimated in relation to the kinetics of other apoptotic parameters such as mitochondrial instability, caspase-3 activation and DNA cleavage.

## Conclusion

In the last decade, a great amount of new information has been generated about the association of LRBA with the immune system, its cellular localization and function. This has improve our understanding about their role in processes such as vesicular trafficking, autophagy and apoptosis. However, many questions remain to be answered. First, is LRBA located in the endomembrane system? Does LRBA form protein complexes with either Rab or Arf? Does LRBA modulate the traffic of receptors such as Fas, NKG2D or NKp46? There is evidence that LRBA prevents the degradation of CTLA-4 by competing AP-1 in human regulatory T lymphocytes, a protein that interacts with Arf1 and Arf6. In addition, the Bchs ortholog gene in *Drosophila* leads to Rab11-dependent vesicular trafficking. This raises the possibility that LRBA modulates several immune receptors by vesicle trafficking and, therefore, it is determinant in the normal development of the immune response.

Secondly, although defects in LRBA lead to poor autophagy, what are the functions of this protein in these cell mechanism? What is its localization in autophagosome, amphisomes and autophagolysomomes? Does LRBA interact physically with autophagy-related molecules such as LC3, ATG or Rab7?, By studying vesicular trafficking and autophagy, we found a possible connection mediated by Rab7, which is needed for the fusion between late endosomes and the autophagolysosome, which might suggest that LRBA is required for the fusion between these two organelles.

Third, can LRBA deficiency affect lysosomal stability and thereby trigger apoptosis? The interaction of LRBA with lysosomes has been described, however, the mechanism underlying its function in lysosomes have not yet been fully elucidated.

**Figure 2:**
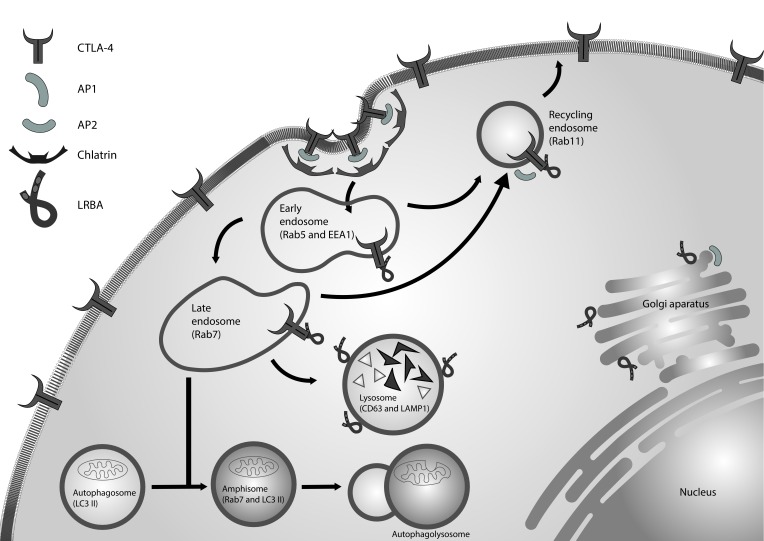
LRBA in the regulation of vesicular trafficking. CTLA-4 is a receptor located in the membrane of activated T lymphocytes. This receptor is internalized in clathrin-coated vesicles by its interaction with AP2. LRBA regulates the recycling of CTLA-4 from endosomes to the cell membrane, preventing its degradation in the lysosomal compartments. The appropriate autophagy influx also need the endocytic vesicles. LRBA is located in these vesicles and might interact with molecules not yet identified to facilitate fusion between the autophagosomes and the late endosomes.

Finally, the association between endosomal trafficking, autophagy, apoptosis in the context of the LRBA function have not yet been addressed. We propose a model for the function of LRBA in the CTLA-4 trafficking (Fig. 2). A complete understanding of the LRBA function also requires a detailed dissection of its structure. It is necessary to characterize the function of their domains and its interaction with other proteins in cellular homeostasis.

New technologies of advanced microscopy, genetic editing such as CRISPR / Cas9 system or next-generation genomics tools, in the context of transcriptomes, proteomes, metabolomes and epigenomes would help to solve many of these questions to clarify the role of this protein and raise strategies for its regulation.

## References

[1] Picard C, Al-Herz W, Bousfiha A, Casanova JL, Chatila T, Conley ME (2015). Primary immunodeficiency diseases an update on the classification from the International Union of Immunological Societies Expert Committee for Primary Immunodeficiency 2015. J Clin Immunol.

[2] Lopez-Herrera G, Tampella G, Pan-Hammarström Q, Herholz P, Trujillo-Vargas CM, Phadwal K (2012). Deleterious mutations in LRBA are associated with a syndrome of immune deficiency and autoimmunity. Am J Hum Genet.

[3] Gamez-Días L, August D, Stepensky P, Revel-Vilk S, Seidel MG, Noriko M (2016). The extended phenotype of LPS-responsive beige-like anchor protein (LRBA) deficiency. J Allergy Clin Immunol.

[4] Lo B, Fritz JM, Su HC, Uzel G, Jordan MB, Lenardo MJ (2016). Blood spotlight CHAI and LATAIE new genetic diseases of CTLA-4 checkpoint insufficiency. Blood.

[5] Alkhairy OK, Abolhassani H, Rezaei N, Fang M, Andersen KK, Chavoshzadeh ZM (2015). Spectrum of phenotypes associated with mutations in LRBA. J Clin Immunol.

[6] Revel-Vilk FU, Keller B, Nabhani S, Gámez-Díaz L, Rensing-Ehl A (2015). Autoimmune lymphoproliferative syndrome-like disease in patients with LRBA mutation. Clin Immunol.

[7] Serwas NK, Kansu A, Santos-Valente E, Kuloglu Z, Demir A, Yaman A (2015). Atypical manifestation of LRBA deficiency with predominant IBD-like phenotype. Inflamm Bowel Dis.

[8] Lo B, Zhang K, Lu W, Zheng L, Zhang Q, Kanellopoulou C (2015). Patients with LRBA deficiency show CTLA4 loss and immune dysregulation responsive to abatacept therapy. Science.

[9] Wang JW, Howson J, Haller E, Kerr WG (2001). Identification of a novel lipopolysaccharide-inducible gene with key features of both A kinase anchor proteins and chs1/beige proteins. J Immunol.

[10] Cullinane AR, Schäffer A, Huizing M (2013). The BEACH Is Hot A LYST of emerging roles for BEACH-domain containing proteins in human disease. Traffic.

[11] Gebauer D, Li J, Jogl G, Shen Y, Myszka DG, Tong L (2004). Crystal structure of the PH-BEACH domains of human LRBA/BGL. Biochemistry.

[12] Jogl G, Gerwald J, Shen Y, Gebauer D, Li J, Wiegmann K (2002). Crystal structure of the BEACH domain reveals an unusual fold and extensive association with a novel PH domain. EMBO J.

[13] Wang J-W, Lockey RF (2014). Lipopolysaccharide-responsive beige-like anchor (LRBA), a novel regulator of human immune disorders. Austin J Clin Immunol.

[14] Wang JW, Gamsby JJ, Highfill SL, Mora LB, Bloom GC, Yeatman TJ (2004). Deregulated expression of LRBA facilitates cancer cell growth. Oncogene.

[15] McCoy KD, Le Gros G (1999). The role of CTLA-4 in the regulation of T cell immune responses. Immunol Cell Biol.

[16] Walker LSK, Sansom DM (2011). The emerging role of CTLA4 as a cell-extrinsic regulator of T cell responses. Nat Rev Immunol.

[17] Valk E, Rudd CE, Schneider H (2008). CTLA-4 trafficking and surface expression. Trends Immunol.

[18] Gamez-Diaz L, Neumann J, Jäger F, Proietti M, Felber F, Soulas-Sprauel P (2017). Immunological phenotype of the murine Lrba knockout. Immunol Cell Biol.

[19] Wee P, Wang Z (2017). Epidermal Growth Factor Receptor Cell Proliferation. Cancers (Basel).

[20] Sigismund S, Argenzio E, Tosoni D, Cavallaro E, Polo S, Di Fiore PP (2008). Clathrin-mediated internalization is essential for sustained EGFR signaling but dispensable for degradation. Dev Cell.

[21] Sorkin A, Goh LK (2008). Endocytosis and intracellular trafficking of ErbBs. Exp Cell Res.

[22] Goh LK, Huang F, Kim W, Gygi S, Sorkin A (2010). Multiple mechanisms collectively regulate clathrin-mediated endocytosis of the epidermal growth factor receptor. J Cell Biol.

[23] Rappoport JZ, Simon SM (2009). Endocytic trafficking of activated EGFR is AP-2 dependent and occurs through preformed clathrin spots. J Cell Sci.

[24] Degli EM, Tour J, Ouasti S, Ivanova S, Matarrese P, Malorni W (2009). Fas death receptor enhances endocytic membrane traffic converging into the Golgi region. Mol Biol Cell.

[25] Lee K, Feig C, Tchikov V, Schickel R, Hallas C, Schütze S (2006). The role of receptor internalization in CD95 signaling. EMBO J.

[26] Park MY, Sudan R, Srivastava N, Neelam S, Youngs C, Wang JW (2016). LRBA is essential for allogeneic responses in bone marrow transplantation. Sci Rep.

[27] Roda-Navarro P, Reyburn HT (2009). The traffic of the NKG2D / Dap10 receptor complex during Natural Killer ( NK ) cell activation. J Biol Chem.

[28] Ogasawara K, Hamerman JA, Hsin H, Chikuma S, Bour-Jordan H, Chen T (2003). Impairment of NK cell function by NKG2D modulation in NOD mice. Immunity.

[29] Engelke M, Pirkuliyeva S, Kühn J, Wong L, Boyken J, Herrmann N (2014). Macromolecular assembly of the adaptor SLP-65 at intracellular vesicles in resting B cells. Sci Signal.

[30] Henriksen L, Grandal MV, Knudsen SL, van Deurs B, Grøvdal LM (2013). Internalization mechanisms of the epidermal growth factor receptor after activation with different ligands. PLoS One.

[31] Shteyn E, Pigati L, Fölsch H (2011). Arf6 regulates AP-1B-dependent sorting in polarized epithelial cells. J Cell Biol.

[32] Khodosh R, Augsburger A, Schwarz TL, Garrity PA (2006). Bchs, a BEACH domain protein, antagonizes Rab11 in synapse morphogenesis and other developmental events. Development.

[33] Gutierrez MG, Munafó DB, Berón W, Colombo MI (2004). Rab7 is required for the normal progression of the autophagic pathway in mammalian cells. J Cell Sci.

[34] Hansen TE, Johansen T (2011). Following autophagy step by step. BMC Biol.

[35] Ryter SW, Mizumura K, Choi AMK (2014). The impact of autophagy on cell death modalities. Int J Cell Biol.

[36] Simonsen A, Birkeland HC, Gillooly DJ, Mizushima N, Kuma A, Yoshimori T (2004). Alfy, a novel FYVE-domain-containing protein associated with protein granules and autophagic membranes. J Cell Sci.

[37] Filimonenko M, Isakson P, Finley KD, Anderson M, Jeong H, Melia TJ (2010). The selective macroautophagic degradation of aggregated proteins requires the PI3P-binding protein Alfy. Mol Cell.

[38] Clausen TH, Lamark T, Isakson P, Finley K, Larsen KB, Brech A (2010). p62/SQSTM1 and ALFY interact to facilitate the formation of p62 bodies/ALIS and their degradation by autophagy. Autophagy.

[39] Ichimura Y, Waguri S, Sou YS, Kageyama S, Hasegawa J, Ishimura R (2013). Phosphorylation of p62 activates the Keap1-Nrf2 pathway during selective autophagy. Mol Cell.

[40] Rahman M, Haberman A, Tracy C, Ray S, Krämer H (2012). Drosophila mauve mutants reveal a role of LYST homologs late in the maturation of phagosomes and autophagosomes. Traffic.

[41] Ravikumar B, Moreau K, Jahreiss L, Puri C, Rubinsztein DC (2010). Plasma membrane contributes to the formation of pre-autophagosomal structures. Nat Cell Biol.

[42] Popovic D, Dikic I (2014). TBC 1D5 and the AP2 complex regulate ATG9 trafficking and initiation of autophagy. EMBO Rep.

[43] Coccia M, Harrison OJ, Schiering C, Asquith MJ, Becher B, Powrie F (2012). IL-1ß mediates chronic intestinal inflammation by promoting the accumulation of IL-17A secreting innate lymphoid cells and CD4+ Th17 cells. J Exp Med.

[44] Saitoh T, Fujita N, Jang MH, Uematsu S, Yang BG, Satoh T (2008). Loss of the autophagy protein Atg16L1 enhances endotoxin-induced IL-1 beta production. Nature.

[45] Plantinga TS, Joosten LAB, Netea MG (2011). ATG16L1 polymorphisms are associated with NOD2-induced hyperinflammation. Autophagy.

[46] Zhou R, Yazdi A, Menu P, Tschopp J (2011). A role for mitochondria in NLRP3 inflammasome activation. Nature.

[47] Nakahira K, Haspel JA, Rathinam VA, Lee SJ, Dolinay T, Lam HC (2011). Autophagy proteins regulate innate immune response by inhibiting NALP3 inflammasome-mediated mitochondrial DNA release. Nat Immunol.

[48] Harris J, Hartman M, Roche C, Zeng SG, O&apos;Shea A, Sharp FA (2011). Autophagy controls IL-1beta secretion by targeting Pro-IL-1beta for degradation. J Biol Chem.

[49] Rathinam VA, Vanaja SK, Fitzgerald KA (2012). Regulation of inflammasome signaling. Nat Immunol.

[50] van Nierop K, Muller FJ, Stap J, Van Noorden CJ, van Eijk M, de Groot C (2006). Lysosomal destabilization contributes to apoptosis of germinal center B-lymphocytes. J Histochem Cytochem.

[51] Repnik U, Stoka V, Turk V, Turk B (2012). Lysosomes and lysosomal cathepsins in cell death. Biochim Biophys Acta.

[52] Yuan XM, Li W, Dalen H, Lotem J, Kama R, Sachs L, Brunk UT (2002). Lysosomal destabilization in p53-induced apoptosis. Proc Natl Acad Sci U S A.

[53] Mukhopadhyay S, Panda PK, Sinha N, Das DN, Bhutia SK (2014). Autophagy and apoptosis Where do they meet?. Apoptosis.

[54] Lemmon MA (2007). Pleckstrin Homology (PH) domains and phosphoinositides. Biochem Soc Symp.

[55] Xu C, Min J (2011). Structure and function of WD40 domain proteins. Protein Cell.

[56] Burgess A, Mornon JP, De saint-basile G, Callebaut I (2009). A concanavalin A-like lectin domain in the CHS1/LYST protein, shared by members of the BEACH family. Bioinformatics.

[57] Tuand K, Stijnen P, Volders K, Declercq J, Nuytens K, Meulemans S (2016). Nuclear localization of the autism candidate gene neurobeachin and functional interaction with the NOTCH1 intracellular domain indicate a role in regulating Transcription. PLoS One.

