# Supplementary material for: LRBA in the endomembrane system
Source: Colomb Med (Cali). 2018 Sep 30;49(3):236–43. doi: 10.25100/cm.v49i2.3802 (PMC6220489; doi:10.25100/cm.v49i2.3802)
Supplement: Related texts

# Review article: LRBA en el sistema de endomembranas

## Introducción

Se han reportado más de 400 genes que afectan cuantitativa y/o cualitativamente y de manera sintomática el sistema inmune ^1^. Entre estos se encuentra *LRBA* (del inglés, *Lipopolysaccharide-responsive and beige-like anchor protein)*; las mutaciones homocigotas o heterocigotas compuestas en este gen, causan pérdida de la expresión de la proteína LRBA, lo que se asocia a un amplio espectro de manifestaciones clínicas e inmunológicas ^1^. El primer reporte sobre la deficiencia de LRBA fue realizado, en cinco pacientes procedentes de cuatro familias consanguíneas con hipogamaglobulinemia, con manifestaciones autoinmunes e infecciones recurrentes, ^2^. Posteriormente se han publicado varios reportes de individuos con deficiencia de la proteína LRBA los cuales presentaron un espectro más amplio de manifestaciones clínicas que incluyeron: una mayor susceptibilidad a las infecciones, poliautoinmunidad, linfoproliferación y gastropatía, todo ello asociado o no a hipogamaglobulinemia ^3^^-^^5^ . También se reportó un individuo asintomático ^6^.

Con relación a los hallazgos inmunológicos, se observó que los linfocitos T de pacientes con deficiencia de LRBA presentaron un aumento en la apoptosis inducida por inanición o estaurosporina (un potente inhibidor de las proteinas-cinasas y activador de las caspasas) y controversialmente, una disminución en la apoptosis inducida por Fas ^7^. Además, se determinó en estas células una disminución en la expresión de CTLA-4 (del inglés *Cytotoxic T-Lymphocyte Antigen 4*), tanto en la membrana como a nivel intracelular ^8^. En los linfocitos B provenientes de pacientes se observó una disminución en la producción de anticuerpos *in vitro*, un incremento en la apoptosis y un defecto en la autofagia tras la inanición ^2^. En conjunto, estos estudios sugieren que LRBA es importante para la respuesta humoral, la regulación inmune y la defensa contra las infecciones, tanto de microorganismos extracelulares como intracelulares, influenciando la supervivencia y la homeostasis de los linfocitos T y B.

## LRBA: características generales

*LRBA* está localizado en el cromosoma 4q31.3, posee un tamaño de 750,839 pb y está organizado en 58 exones. La proteína posee 2,863 aminoácidos con un peso aproximado de ~319 KDa (Fig. 1). En el ratón, se detectaron tres diferentes transcriptos de 1344, 836 y 787 pb que se expresan diferencialmente en los tejidos ^9^. Adicionalmente, la expresión del ARN mensajero (ARNm) de *LRBA* se incrementó entre dos y cuatro veces luego de la estimulación con lipopolisacárido (LPS) en líneas celulares ^9^.

**Figura 1:** Representación esquemática del LRBA y la proteína que codifica. El LRBA humano contiene 750,839 pb organizado en 58 exones. Su ubicación citogenética es 4q31.3. La proteína humana LRBA tiene 2,863 aminoácidos compuestos de múltiples dominios. El peso molecular predicho es de 319 kD.

Esta proteína posee diferentes dominios, entre ellos el más caracterizado es el dominio BEACH (Del inglés, *beige* and Chediak-Higashi). Este dominio, de aproximadamente ~280 aminoácidos, se conserva en varias proteínas agrupadas como familia BDCP (Del inglés*, BEACH domain containing proteins*) ^10^, en las cuales se localiza generalmente hacia el extremo C-terminal ^(^^11^^,^^12^. La función de este dominio es desconocida y su secuencia no presenta homología con ninguna proteína en la base de datos del proteoma humano ^(^^11^^,^^12^. Además del dominio BEACH, las BDCP comparten los dominios ConA-like (del inglés, *concanavalin A-like*), PH (del inglés, *Pleckstrin homology*) y WD40 (repeticiones de triptófano y ácido aspártico. Por su parte, el dominio DUF1088 (del inglés, *domain of unknown function 1088*) es compartido solo por LRBA y su parálogo Neurobeachin, lo cual podría indicar una función específica de estas dos proteínas no compartida por otros miembros de las BDCP.

Además de estos dominios, LRBA parece contener un dominio VHS (del inglés, *VPS* (*vacuolar protein sorting*)*-27, Hrs* (*hepatocyte growth factor-regulated tyrosine kinase sustrate*) *STAM* (*signal transducing adaptor molecule*) ^(^^13^. Las posibles funciones de estos dominios se explican en la Tabla 1. 

**Tabla 1:** Características generales de los dominios de la proteín LRBA.

Dominio	Características	Referencia
BEACH	Está presente en proteínas implicadas en el tráfico de vesículas, dinámica de membranas y señalización de receptores.	^12^^,^^13^
PH	Interactúa con fosfatidil-inositol dentro de membranas biológicas, proteínas G hetero-triméricas y proteína quinasa C. Esto permite el direccionamiento apropiado de proteínas a diferentes compartimentos celulares o la activación de vías de transducción de señales.	^54^
WD40	Implicado en transducción de señales, control del ciclo celular y la apoptosis. Sitio para la interacción transitoria proteína-proteína o para el ensamblaje de complejos proteicos.	^55^
ConA-like	Participa en el tráfico y distribución de proteínas por vías secretoras.	^56^
DUF1088	La función de este dominio es desconocida. Sin embargo, una secuencia de penta-arginina localizada en el dominio DUF1088 de Neurobeachin fue identificada como una señal potencial de localización nuclear.	^57^
VHS	Encontrado en la porción N terminal de proteínas asociadas con endocitosis y/o tráfico vesicular. Posiblemente funciona como un dominio adaptador que ayuda a localizar las proteínas en la membrana celular o en membranas de la maquinaria endocítica.	^13^

Con respecto a la expresión específica de tejido, se han observado transcriptos de esta proteína principalmente en células de médula ósea, nódulo linfático, bazo, hígado fetal, placenta, riñón y páncreas por medio de RT-PCR (del inglés, *Reverse transcription polymerase chain reaction*) y qPCR (del inglés, *quantitative polymerase chain reaction*) ^2^. De manera interesante, la expresión de esta proteína no se limita a tejidos del sistema inmune, e incluso su expresión se ve considerablemente aumentada en tejidos provenientes de varios tipos de cáncer ^14^. Estos hallazgos sugieren que la función de LRBA está más relacionada con mecanismos celulares básicos de crecimiento, desarrollo y supervivencia. A nivel subcelular, utilizando una sonda dirigida contra el dominio BEACH de LRBA, se observó esta proteína en el citosol, aparato de Golgi y algunos lisosomas en la línea celular de macrófagos murinos RAW264.7 estimulados con LPS ^9^. Además, con la utilización del microscopio electrónico, se observó que LRBA se localizó en el retículo endoplásmico, la membrana plasmática y las vesículas de endocitosis asociadas a clatrina en estas mismas células ^9^.

## Función del LRBA

### LRBA participa en el tráfico vesicular y reciclaje de receptores.

CTLA-4 (Del inglés *Cytotoxic T-Lymphocyte Antigen* 4) es un receptor proteico situado en la membrana celular de los linfocitos T activados. Su función es regular la homeostasis y la tolerancia inmunológica periférica inhibiendo la activación de los linfocitos T, por competición con la molécula coestimuladora CD28, mediante la unión a los ligandos CD80 y CD86, los cuales se expresan en la superficie de las células presentadoras de antígeno ^15^. Mientras que CD28 reside principalmente en la superficie celular, CTLA-4 se localiza primero en los compartimentos intracelulares como el aparato de Golgi, los endosomas, los gránulos secretores y las vesículas lisosomales. La estimulación del TCR (del inglés, *T cell receptor*) promueve el tráfico de CTLA-4 desde los compartimentos vesiculares hacia la membrana celular, sin embargo, en la membrana, este receptor es continuamente endocitado dentro de las vesículas cubiertas de clatrina. Una vez endocitadas, algunas moléculas de CTLA-4 son recicladas a la membrana celular, mientras que otras son rápidamente degradadas en los compartimentos lisosomales ^16^^,^^17^. CTLA-4 contiene una pequeña cola citoplasmática con dos motivos de tirosina en la posición Y_201_VKM y Y_218_FIP. Diversas proteínas intracelulares se unen al motivo Y_201_VKM, incluyendo AP-1 y AP-2 (del inglés *clathrin-associated adaptor*). AP-2 media la internalización de CTLA-4 desde la superficie celular a los endosomas y los compartimentos lisosomales, mientras que AP-1 regula en el tráfico de CTLA-4 desde el aparato de Golgi a los compartimentos endosomales y lisosomas para su degradación ^17^. En un estudio reciente, se demostró que la proteína LRBA es necesaria para el tráfico vesicular de CTLA-4 hacia la membrana plasmática ^8^. Cuando CTLA-4 es requerida en la membrana, LRBA regula el reciclaje de CTLA-4 desde los endosomas a la membrana celular ayudando a mantener los niveles intracelulares de CTLA-4 para su inmediata movilización a la membrana. De esta manera, una deficiencia de LRBA favorece una rápida degradación de CTLA-4 en los compartimentos lisosomales, con su consecuente disminución tanto intracelular como extracelularmente ^8^. Este trabajo también demostró que LRBA interactúa por medio del dominio ConA-*like* y el dominio PH-BEACH con la cola citoplasmática de CTLA-4, específicamente con el motivo Y_201_VKM. Finalmente, el silenciamiento de AP-1, pero no AP-2 puede rescatar parcialmente la pérdida CTLA-4 en las células deficientes de LRBA. Estos resultados sugieren que LRBA bloquea el tráfico de CTLA-4 a los lisosomas, compitiendo con AP-1 por la unión a este motivo ^8^. Hallazgos recientes indicaron que los linfocitos CD4+ Foxp3+ provenientes de células del bazo de ratones deficientes para LRBA también exhiben una disminución intracelular de CTLA-4 ^18^.

Otro estudio que ilustró el papel de LRBA en el tráfico vesicular fue realizado con el receptor del factor de crecimiento epidermal (EGFR, del inglés, *epidermal growth factor receptor*). Mutantes dominantes negativas para LRBA disminuyeron la expresión y fosforilación de EGFR ^14^. El EGFR es un receptor tirosina kinasa el cual se expresa en una gran variedad de tejidos, interactuando no sólo con el EGF sino también con TGF-( (Del inglés, *Transforming growth factor-(*), y otros ligandos. Esto induce la dimerización y trans-auto-fosforilación del receptor, reclutando proteínas que activan las vías de señalización necesarias para la proliferación, la diferenciación, el crecimiento, la migración e la inhibición de la apoptosis ^19^. En los linfocitos, al igual que CTLA-4, su expresión en la superficie celular es regulada por la endocitosis mediada por clatrina, lo cual conduce ya sea a su degradación por vía lisosomal o a su reciclaje a la superficie celular ^20^^,^^21^. Defectos en el tráfico vesicular de este receptor resultan en una localización aberrante, lo cual potencia la señalización permitiendo el desarrollo de cáncer ^22^. Aunque no se conoce si LRBA y EGFR interactúan físicamente, se ha comprobado que AP-2 facilita la endocitosis y el tráfico de EGFR por la vía endocítica ^3^.

Finalmente, en pacientes deficientes de LRBA se demostró una disminución en la apoptosis mediada por el receptor de muerte Fas, cuando se comparó con los individuos sanos ^6^. El receptor Fas promueve la apoptosis por la vía extrínseca, después de la unión al ligando de Fas (FasL). Posterior a la unión Fas/FasL, se da un proceso de internalización del receptor, similar a lo que ocurre con EGFR, el cual es crucial para desencadenar la apoptosis ^24^. Hasta la fecha, se desconoce si existen alteraciones en la cantidad de receptores Fas en la membrana celular en pacientes con deficiencia en LRBA, pero el incremento de FasL en suero en dichos pacientes podría sugerir que posiblemente la deficiencia de LRBA tenga un efecto en el receptor Fas similar al de CTLA-4, regulando su tráfico ya sea hacia la membrana celular o lisosomas ^25^. 

Estas evidencias nos llevan también a preguntarnos si LRBA estaría implicado en el tráfico vesicular de otros receptores en linfocitos?. En ratones deficientes para LRBA se ha reportaron defectos en la fosforilación de ERK1/2 (del inglés, *extracellular signal-regulated kinases*) y AKT en células NK (del inglés, *natural killer*) estimuladas con anticuerpos anti-NKG2D o anti-NKp46, con una disminución significativa en la producción de IFN-gamma después de estos estímulos ^26^. Aunque en este estudio no se determinaron los niveles de expresión de estos receptores en los ratones deficientes de LRBA, existen algunas evidencias que indican que la exposición a sus ligandos específicos, induce la degradación de NKG2D y DAP10, la molécula adaptadora intracelular de este receptor en los lisosomas ^27^. Esta regulación parece ser mediada por clatrina ^28^. No se conoce si LRBA está involucrado en el tráfico intracelular de estos receptores en células NK. De la misma manera, considerando que la deficiencia de LRBA conlleva a defectos en las respuestas de anticuerpos, es importante considerar también, si esta molécula regula la expresión y función de otros receptores cuya expresión es modulada por compartimentos vesiculares, procesos determinantes para la activación del linfocito B ^29^. 

A pesar de los estudios sobre la modulación de algunos receptores celulares mediada por LRBA, es poco lo que se conoce sobre las vías de tráfico intracelular utilizadas por esta molécula para realizar dicha función. Se ha descrito que dicho transporte se realiza mediante el reclutamiento de proteínas en vesículas recubiertas por clatrina, en el cual el receptor es removido a endosomas tempranos y posteriormente degradado por enzimas lisosomales o reciclado a la superficie celular ^30^. AP-1 se considera necesario para este transporte. La localización de AP-1 en el aparato de Golgi y en endosomas, depende de su unión directa a Arf (del inglés: *ADP-ribosylation factor*)-1 y específicamente en endosomas de reciclaje, a Arf-6, entre otros complejos proteicos ^31^. Como ya se mencionó, LRBA inhibe la degradación de CTLA-4 en lisosomas por competición con AP-1, demostrando ser necesario para la movilización a estos compartimientos ^8^. Sin embargo, la relación de LRBA con toda la vía endosomal no es clara. El reciclaje de receptores depende además de los compartimientos vesiculares, de proteínas involucradas incluidas las Arf, de enzimas como la fosfolipasa D y de las proteínas Rab. De manera interesante, un estudio realizado en *Drosophila*, sugiere una posible interacción funcional entre bchs (del inglés *blue cheese*), un miembro de la familia BEACH, y Rab11 ^32^. Estos hallazgos sugieren que, así como Rab11, Bchs podría ser un regulador del tráfico de las vesículas. 

Por lo tanto, son necesarios estudios que cuantifiquen la expresión de los receptores mencionados en células deficientes de LRBA (silenciadas o provenientes de pacientes). También sería útil el uso de técnicas avanzadas de microscopía, con lo cual se pudiera demostrar la localización de LRBA en otras organelas involucradas en este tipo de tráfico. Además, estudios de co-inmunoprecipitación entre LRBA y las proteínas de tráfico vesicular, el uso de drogas que bloqueen este tráfico o la implementación de dominantes negativos de ARFs, fosfolipasa D o las Rab, podrían ampliar nuestro conocimiento sobre la interacción de LRBA con otras moléculas de tráfico vesicular y así determinar su relación con la expresión en membrana de receptores como EGFR, Fas o los receptores activadores de las células NK, linfocitos B u otros en células inmunes.

### LRBA es una molécula implicada en autofagia

La autofagia es un mecanismo de degradación lisosomal por el cual la célula destruye organelas dañadas y microorganismos y ayuda a reciclar macromoléculas. En este proceso se forma una doble membrana de aislamiento o fagóforo cerca al blanco, que luego se elonga, rodeándolo, para posteriormente madurar con la asimilación de una carga citosólica formando el autofagosoma. Para que la maduración sea efectiva, el autofagosoma se debe fusionar con los endosomas tempranos o tardíos formando una estructura llamada anfisoma ^33^^,^^34^. Por último, el autofagosoma maduro se fusiona con el lisosoma para permitir la degradación de su contenido por las proteasas lisosómicas y enzimas hidrolíticas ^35^.

Cuando estudiamos los genes homólogos de *LRBA*, encontramos que varios de ellos están implicados en la autofagia. *WDFY3* (del inglés - *WD and FYVE zinc finger domain containing protein 3*), codifica una proteína que colocaliza con marcadores de autofagosomas ^36^^,^^37^. Además, su dominio PH-BEACH interacciona con la proteína p62 ^34^. P62 es una proteína localizada en los sitios de formación de los autofagosomas y se asocia tanto con LC3 (Del inglés, *microtubule-associated protein 1A/1B-light chain 3*) como con proteínas ubiquitinadas ^34^^,^^38^^,^^39^. LC3 es una proteína citosólica que cuando se conjuga con una fosfatidiletanolamina es reclutada a la membrana del autofagosoma favoreciendo el flujo autofágico ^34^. Por otro lado, defectos en el gen ortólogo de *LYST*, mauve (mv) en *Drosophila*, afecta la función de autofagosomas ^40^. 

En células B inmortalizadas provenientes de pacientes con deficiencia de LRBA se observó una reducción significativa en el proceso de autofagia en respuesta a la inanición, además hubo un incremento en el área del aparato de Golgi, una acumulación de los autofagosomas y la presencia de centriolos, al compararse con las células provenientes de un individuo sano ^2^. Estos resultados sugieren que el flujo de la autofagia está deteriorado en la deficiencia de LRBA, un hallazgo que fue demostrado por una fusión deficiente del autofagosoma a los lisosomas en linfocitos B inmortalizados de pacientes deficientes de LRBA, sometidos a inanición ^2^. Sin embargo, ¿cuál son las posibles funciones de LRBA en los procesos de autofagia? Considerando que tanto el tráfico de receptores de superficie celular como la autofagia necesitan de vesículas endocíticas, y que LRBA posiblemente se encuentra ubicado en estas vesículas, una posible hipótesis es que LRBA facilite la fusión entre el autofagosoma y el endosoma tardío y, por consiguiente, la formación del anfisoma. Uno de los marcadores de los endosomas tardíos es Rab7 y se ha demostrado en células CHO transfectadas con la mutante dominante negativa de Rab7, T22N, que esta molécula se requiere para la formación de los anfisomas ^33^. Otra hipótesis que se podría considerar es que, de la misma manera que con el tráfico vesicular de CTLA-4, también en el contexto de autofagia en linfocitos, LRBA compita con proteínas adaptadoras de clatrina, regulando este proceso. Esto ya que se ha observado, que moléculas como clatrina y sus proteínas adaptadoras son también importantes para el flujo autofágico ^41^^,^^42^. Por otra parte, herramientas *in silico* han predicho que LRBA interactúa con LC3 ^13^.

En un intento por asociar los defectos en la autofagia con las características clínicas encontradas en los individuos con deficiencia de LRBA, es importante tener en cuenta que una de las manifestaciones clínicas más comunes entre estos individuos es la gastropatía, en muchos casos expresada como Enfermedad Inflamatoria Intestinal (EII) o enfermedad similar a EII, una condición que se caracteriza por una inflamación recurrente destructiva en el tracto intestinal ^3^^-^^5^. Un incremento en la producción de IL-1β en la mucosa intersticial se ha involucrado con la inflamación descontrolada en esta enfermedad ^43^. Los macrófagos derivados de ratones deficientes para *ATG16L1* (del inglés, *autophagy-related 16-like 1*), los cuales presentan un defecto en la autofagia, muestran una elevada producción de IL-1β después de la estimulación con LPS ^44^. De la misma manera, el polimorfismo rs2241880 en *ATG16L1* incrementó la producción de IL-1β en células mononucleares de sangre periférica aisladas de pacientes con EII ^45^. La autofagia puede regular la producción de IL-1β mediante dos vías: en ausencia de autofagia, las mitocondrias defectuosas se acumulan generando un incremento de ROS (del inglés, *reactive oxygen species*) y liberando ADN mitocondrial al citoplasma, eventos que activan el inflamosoma, aumentando la producción de IL-1β ^46^^,^^47^. Por otra parte, la degradación de la pro-IL-1β mediada por los procesos de autofagia limita el sustrato disponible para el inflamasoma, regulando de esta manera la activación de la IL-1β ^48^^,^^49^. Por lo tanto, las alteraciones en la autofagia limitarían este mecanismo, aumentando los niveles de IL-1β e induciendo inflamación. No se conoce si las manifestaciones intestinales en los pacientes con deficiencia en LRBA están mediadas por una excesiva actividad del inflamosoma y la exagerada producción de IL-1β. 

Se requieren estudios de microscopia con células deficientes de LRBA que estudien la distribución intracelular de moléculas como Rab7 y LC3 tras el flujo autofágico. Sería interesante también investigar si LRBA interactúa físicamente con alguna de las moléculas Rab involucradas en los diferentes tipos de autofagia, por medio de ensayos de co-inmunoprecipitación. El papel de LRBA en la actividad del inflamosoma pudiera abordarse mediante la medición de la producción de IL-1β en células fagocíticas o del epitelio intestinal deficientes de LRBA o determinando la actividad del auto-inflamosoma activo por medio de redistribución de ASC (del inglés, *apoptosis-associated speck-like protein containing a caspase-recruitment domain*), la cual es una proteína que recluta las caspasas necesarias en para la activación del inflamosoma. 

### LRBA participa en la apoptosis

En linfocitos B inmortalizados sometidos a inanición provenientes de pacientes deficientes de LRBA, se observó un incremento significativo de la apoptosis ^2^. Adicionalmente, en células HeLa silenciadas para LRBA expuestas a luz ultravioleta o estaurosporina, se observó un mayor clivaje de PARP (por sus siglas en inglés, *Poly (ADP-ribose) polymerase*) y de la caspasa 3, eventos necesarios para iniciar la cascada de señalización que finalmente lleva a la apoptosis ^14^.

La apoptosis es un tipo de muerte celular programada por la misma célula con el fin de controlar su desarrollo y crecimiento. Por mucho tiempo se ha considerado a las caspasas como las proteasas involucradas en la ejecución del programa de apoptosis, y hoy en día se conoce que la desestabilización parcial de la membrana lisosomal, con la consecuente liberación de enzimas hidrolíticas, puede iniciar y ejecutar el programa de apoptosis, activando las caspasas ^50^^,^^51^. En los centros germinales provenientes de amígdalas se observó que la desestabilización lisosomal en linfocitos B contribuye a la apoptosis ^50^. La inducción del daño lisosomal en estos linfocitos B resultó en el clivaje de la caspasa 8 y también del ADN, debido a la actividad incrementada de la catepsina B en el citoplasma. De manera interesante, se ha observado que p53 induce desestabilización lisosomal ^52^ y el promotor de *LRBA* es regulado negativamente por p53 ^14^. *P53* es un gen supresor de tumores que codifica un factor de transcripción nuclear cuya expresión se incrementa en respuesta al daño, deteniendo el ciclo celular y facilitando la reparación del ADN o induciendo apoptosis ^52^. Se podría plantear la hipótesis que en ausencia de *LRBA*, p53 induce desestabilización lisosomal y como consecuencia la apoptosis en linfocitos. Sin embargo, hasta el momento no se conoce el mecanismo por el cual p53 induce la desestabilización lisosomal ni como LRBA protege las células de la apoptosis. 

No obstante, parece existir una conexión entre los mecanismos de autofagia y la apoptosis. Aunque la diafonía entre los procesos de autofagia y la apoptosis es compleja también es crítica para el destino celular. Bajo ciertas condiciones celulares, la autofagia puede promover la supervivencia celular y evitar la apoptosis ^53^. Por ejemplo, el aumento de la autofagia en células privadas de nutrientes o factores de crecimiento permite la supervivencia celular al inhibir la apoptosis. Un mecanismo sugerido por el cual la autofagia inhibe la apoptosis, es el secuestro de las mitocondrias dañadas por el autofagosoma, evitando así que el citocromo c mitocondrial liberado sea capaz de formar un apoptosoma funcional en el citoplasma ^53^. Sin embargo, en otras condiciones, la autofagia puede culminar en la muerte celular, de manera dependiente o independiente de la apoptosis ^53^. Adicionalmente, son varias las proteínas que comparten un papel dual tanto en la apoptosis como en la autofagia ^53^. Estas proteínas autofágicas regulan positivamente o negativamente la apoptosis mitocondrial directa o indirectamente a través del clivaje de proteasas ^53^. En el contexto de las células deficientes de LRBA, se ha demostrado una autofagia defectuosa con un aumento en la apoptosis. No se conoce si una alteración en la depuración de las mitocondrias dañadas o un exagerado clivaje de las proteasas son los responsables de este fenotipo celular. 

Por lo tanto, es necesario evaluar la cantidad y la integridad de los lisosomas en las células inmunes deficientes de LRBA. Además, se pudiera estimar la cinética de desestabilización lisosomal en relación con la cinética de otros parámetros apoptóticos como, inestabilidad mitocondrial, activación de caspasa-3 y escisión de ADN.

## Conclusión 

En la última década se ha generado una gran cantidad de nueva información sobre la asociación de LRBA con el sistema inmune, su localización celular y su función. Esto ha impulsado nuestra comprensión de su papel en los procesos como el tráfico vesicular, la autofagia y la apoptosis. Sin embargo, muchas preguntas quedan por responder. Primero, ¿Está LRBA localizado en el sistema de endomembranas? ¿Forma LRBA complejos proteicos con otras proteínas como las Rab o las Arf? ¿modula LRBA el tráfico de receptores como Fas, NKG2D o NKp46? Hay evidencia de que LRBA evita la degradación de CTLA-4 por competición de AP-1 en linfocitos T reguladores humanos, una proteína que interactúa con Arf1 y Arf6. Además, el gen ortólogo *Bchs* en *Drosophila* modela el tráfico dependiente de Rab11. Esto plantea la posibilidad de que LRBA module varios receptores inmunes mediante el tráfico de vesículas y, por lo tanto, sea determinante en el desarrollo normal de la respuesta inmune. 

En segundo lugar, aunque se ha determinado que los defectos en LRBA conlleva a una autofagia deficiente, ¿cuáles son las funciones de esta proteína en estos procesos?, ¿Cuál es su localización en autofagosoma, anfisomas, autofagolisomas?, ¿interactua LRBA físicamente con moléculas implicadas en la autofagia como LC3, ATG o Rab7?, Al estudiar el tráfico vesicular y la autofagia encontramos una conexión medida por Rab7, el cual se necesita para la fusión entre los endosomas tardíos y el autofagolisosoma, por lo que es posible pensar que LRBA puede ser requerido para la fusión entre estas dos organelas. 

En tercer lugar, ¿puede la deficiencia de LRBA afectar la estabilidad lisosomal y de este modo desencadenar la apoptosis? Se sabe de la interacción de LRBA con estas vesículas, sin embargo, los mecanismos que subyacen a su función en este contexto, aún no se han dilucidado por completo. 

Finalmente, la asociación entre el tráfico endosomal, la autofagia, la apoptosis y la proteína LRBA aún no se han abordado. Sin embargo, con base a lo expuesto en esta revisión nosotros proponemos un modelo de LRBA en el tráfico de CTLA-4 (Fig. 2). Una comprensión completa de la función LRBA también requiere una disección detallada de su estructura. Es necesario caracterizar la función de sus dominios en la interacción con otras proteínas y en general, en la homeostasis celular.

**Figura 2:** LRBA en la regulación del tráfico vesicular.CTLA-4 es un receptor situado en la membrana de los linfocitos T activados. Este receptor es internalizado en vesículas recubiertas de clatrina mediante su interacción con AP2. LRBA regula el reciclaje de CTLA-4 desde los endosomas a la membrana celular, impidiendo su degradación en los compartimentos lisosomales. Los procesos de autofagia también necesitan de las vesículas endocíticas. LRBA se encuentra ubicado en estas vesículas y podría interaccionar con moléculas aún no identificadas para facilitar la fusión entre el autofagosoma y el endosoma tardío.

Se espera que las nuevas tecnologías de microscopia avanzada, de edición genética como CRISPR/Cas9 o la genómica, y los nuevos conceptos como transcriptoma, proteoma, metaboloma, epigenoma, ayuden a resolver muchas de estas preguntas para delucidar el papel de esta proteína y plantear estrategias para su regulación.
